# Development of Data-Driven Models for Just-in-Time Digital Self-Management Advice to Improve Physical Functioning in Hip and Knee Osteoarthritis: Protocol for the e-cOAch Cross-Over Study

**DOI:** 10.2196/94709

**Published:** 2026-06-22

**Authors:** Femke Groen, Mariëtte de Rooij, Corelien J J Kloek, Cindy Veenhof, Michel C A Klein, Abdallah Al-Janabi, Erik S van Haeringen, Amber Ronteltap, Bas Paans-Cijs, Britt van Dongen, Dorien E Ginsel, Di-Janne A Barten, Thomas J Hoogeboom, Martijn F Pisters

**Affiliations:** 1 Department of Rehabilitation, Physiotherapy Science and Sports, UMC Utrecht Brain Center, University Medical Center Utrecht, Heidelberglaan 100, Utrecht, Utrecht, 3584 CX, The Netherlands, 31 887555555; 2 Center for Rehabilitation and Rheumatology, Reade, Amsterdam, North Holland, The Netherlands; 3 Research Group Innovation of Human Movement Care, University of Applied Sciences Utrecht, Utrecht, Utrecht, The Netherlands; 4 Department of Computer Science, Vrije Universiteit Amsterdam, Amsterdam, North Holland, The Netherlands; 5 Department of Health Promotion, Maastricht University, Maastricht, Limburg, The Netherlands; 6 Department of Health Innovations & Technology, Fontys University of Applied Sciences, Eindhoven, North Brabant, The Netherlands

**Keywords:** osteoarthritis, self-management, artificial intelligence, physical functioning, lifestyle modification, physical activity, weight management, sleep management

## Abstract

**Background:**

Clinical guidelines recommend a stepped-care strategy for patients with hip and knee osteoarthritis that begins with nonoperative approaches, including education, pain medication, and self-care. However, the implementation of stepped-care remains limited. Digital self-management interventions have the potential to support patients in applying lifestyle advice and self-care strategies, but current tools often provide generic support without long-term continuity. Artificial intelligence offers new opportunities to deliver personalized, just-in-time self-management interventions for people with osteoarthritis. The development of such artificial intelligence algorithms is limited due to a lack of rich, longitudinal datasets.

**Objective:**

The primary objective of this study is to develop and evaluate data-driven models that support personalized recommendations on the optimal timing and optimal advice (physical activity promotion, sleep optimization, weight management, no program) for individuals with hip or knee osteoarthritis.

**Methods:**

This prospective cross-over study aims to include 600 people with hip or knee osteoarthritis, meeting the National Institute for Health and Care Excellence criteria. The study is registered at ClinicalTrials.gov (registered February 2, 2026, NCT07423858). We aim to screen digital health literacy in our sample. Participants will be recruited across the Netherlands and will use the e-cOAch web app, with 3 self-care programs (ie, physical activity promotion, weight management, and sleep optimization). Each participant will complete all three 12-week programs and one 12-week control period in a randomized sequence. Participants will be followed for 12 months, with biweekly assessments conducted via the app. The primary outcome for model development will be deterioration in physical functioning, measured by a decrease in the Hip Disability and Osteoarthritis Outcome Score subscale activities of daily living of 6.7 or the Knee Injury and Osteoarthritis Outcome Score subscale activities of daily living of 8.2. Secondary outcomes will be pain and participation. Additional measures will include patient characteristics (date of birth, sex, height, level of education, comorbidity, ethnicity, use of a walking device, use of pain medication, device for e-cOAch, duration of osteoarthritis complaints, health and digital literacy, smoking, alcohol use), physical activity, sleep quality and insomnia, psychosocial factors, behavioral determinants, and engagement with the app. These data will inform the development of data-driven models using supervised (causal) machine learning.

**Results:**

The funding for the study was granted in 2023. At the time of manuscript submission, 520 participants had been recruited. Recruitment is expected to be completed in March 2026, with data collection projected to conclude in April 2027. The publication of the results is anticipated in spring 2028.

**Conclusions:**

This study will provide data-driven models that forecast changes in physical functioning over time and support personalized recommendations on the optimal timing of specific self-care programs for people with hip or knee osteoarthritis. These models will be integrated into a new iteration of a self-management app, e-cOAch (version 2), to provide personalized support for people with osteoarthritis across varying levels of digital health literacy.

## Introduction

Osteoarthritis is highly prevalent, affecting roughly 7.7% of the global population in 2021 [1]. Its prevalence is expected to rise substantially by 2050 due to aging populations and rising obesity rates [1]. The hip and knee are 2 commonly affected joints [2]. People with hip or knee osteoarthritis commonly experience pain and stiffness, as well as reduced physical functioning and participation in everyday activities [3,4].

To reduce the impact of osteoarthritis on physical functioning and participation, clinical guidelines for osteoarthritis recommend adopting a stepped-care approach [5,6]. This approach starts with nonoperative strategies, including lifestyle advice and the promotion of self-management. Self-management involves actively managing symptoms, treatment, and lifestyle choices [7]. If this first step is insufficient, nonsurgical treatments, such as guided exercise therapy and dietary support, are recommended. Specialized or multidisciplinary care, including surgery, is considered when earlier steps fail to improve osteoarthritis symptoms adequately [8]. Despite these clear guidelines, the implementation of stepped care in the Netherlands remains limited. Approximately 70% of patients consult an orthopedic surgeon without prior physiotherapy [9].

Improving stepped care implementation and supporting self-management in people with osteoarthritis requires individually tailored support at each step of care [10,11]. Digital health technologies, including wearable activity trackers and connected apps, can support osteoarthritis self-management by facilitating the continuous collection of questionnaires and sensor data [12]. Digital health technologies can, therefore, serve as an effective first-choice tool for self-management support in osteoarthritis-relevant topics, such as lifestyle behaviors and osteoarthritis knowledge [13,14]. Digital delivery allows wide accessibility and flexible use [15]. However, part of the population, with limited health or digital literacy, may face difficulties using digital health technologies. Despite their potential to support osteoarthritis self-management, current interventions lack individually tailored support [14].

The use of artificial intelligence (AI) provides new opportunities to deliver personalized, just-in-time self-management interventions. Continuous data collection through apps and wearable devices can inform AI algorithms that adapt interventions to each person’s status and needs [16]. Machine learning techniques can be used to predict the optimal timing of interventions, while causal inference approaches may help identify which type of self-management advice is most likely to improve pain, physical functioning, and participation [16]. Together, these AI approaches have the potential to make self-management support highly responsive and targeted and therefore optimally effective. However, osteoarthritis-specific AI algorithms remain scarce, largely due to the lack of rich, longitudinal datasets that capture symptoms, behavioral factors, and contextual information over time.

Developing osteoarthritis-specific models that support long-term self-management requires strategies to anticipate changes in symptoms and personalize advice. To ensure that these models represent the broader osteoarthritis population and do not unintentionally increase health disparities, we make an effort to include individuals with diverse levels of digital health literacy. Therefore, the primary objective of this study is to develop and evaluate data-driven models that support personalized recommendations on the optimal timing and optimal advice (physical activity promotion, sleep optimization, weight management, no program) for individuals with hip or knee osteoarthritis. In other words, the study aims to identify an optimal dynamic treatment regime. These models are intended to be integrated into a new iteration of a self-management app, e-cOAch (version 2), to provide personalized support for people with osteoarthritis across varying levels of digital health literacy.

## Methods

### Study Design

**Figure 1.**
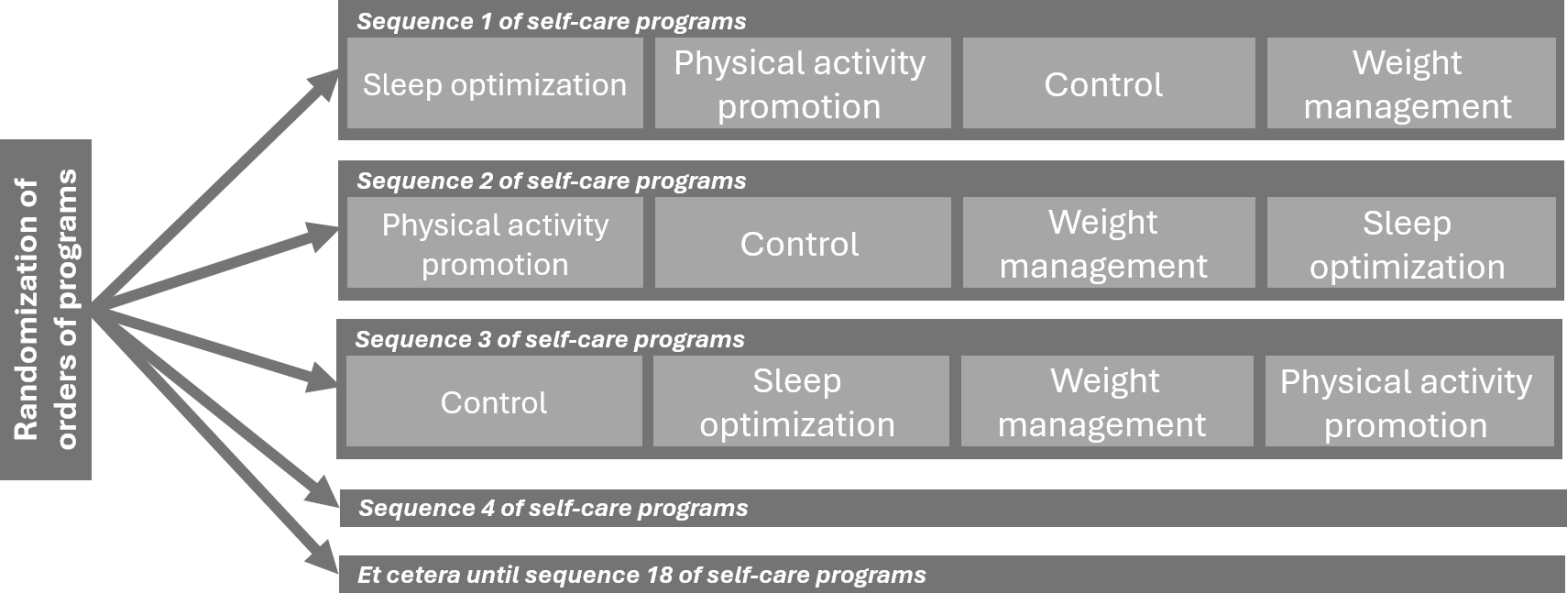
Randomization of orders of self-care programs (sleep optimization, physical activity promotion, weight management) and control.

This is a prospective cross-over study including participants with hip or knee osteoarthritis. Recruitment is planned from September 2025 to March 2026 in the Netherlands. Each participant will be followed for 12 months. Assessments occur every 2 weeks (Figure 1). Participants will complete 3 self-care programs (physical activity promotion, sleep optimization, and weight management) and 1 control period in random order. Programs and control will start in weeks 3, 15, 27, and 39, each lasting 12 weeks. No washout period is included. This study has a cross-over design that allows the evaluation of behavioral interventions in a real-world context. Rather than assuming a return to baseline, the design captures the sustained and cumulative nature of behavior change [17]. This aligns with the objective of identifying an optimal sequencing of self-care programs over time. Recent methodological work by Kulnik et al [17] and Shi et al [18] supports that such designs remain valid in the presence of behavioral carryover effects when appropriately addressed analytically. This study has been reviewed and approved by the NedMec Medical Ethical Committee (NL87119.041.24) and is registered at the International Clinical Trials Registry (registered on May 21, 2025, NL-OMON57573) and ClinicalTrials.gov (registered February 20, 2026, NCT07423858). This protocol was developed in accordance with the SPIRIT (Standard Protocol Items: Recommendations for Interventional Trials) checklist [19] and the TRIPOD (Transparent Reporting of a Multivariable Prediction Model for Individual Prognosis or Diagnosis) + AI checklist.

### Participants

We aim to include 600 participants through a broad recruitment strategy across multiple channels to reach Dutch individuals with osteoarthritis. Eligibility is based on the criteria listed in Textbox 1. Participants must be adults with self-administered osteoarthritis according to the National Institute for Health and Care Excellence criteria [10]; key exclusion criteria include systemic arthritis or recent or upcoming lower limb surgery.

Textbox 1. **Eligibility criteria.**

**Inclusion criteria**

- Have a hip and/or knee joint that, self-administered through a questionnaire, meets the National Institute for Health and Care Excellence clinical criteria for osteoarthritis [10]:
  1. Aged 45 years or over
  2. Activity-related pain at the joint
  3. Joint morning stiffness that lasts no longer than 30 minutes or no morning stiffness at the joint

- History of pain at the joint for at least 3 months
- Have access to a smartphone with internet connection and an email address
- Able to give informed consent and willing to commit to all study evaluation and assessment procedures
- Able to read and understand texts in Dutch at the B1 language level

**Exclusion criteria**

- Self-reported systemic arthritis (eg, rheumatoid arthritis, gout) avoids confounding due to overlapping symptoms
- Scheduled for lower limb joint surgery within the next year, as surgical interventions could affect outcomes and confound the assessment of treatment effects
- Underwent lower limb joint surgery (total hip, total knee) in the last year, as recent surgery may alter symptoms and confound the assessment of treatment effects

### Recruitment

Recruitment is coordinated by the University Medical Center Utrecht and supported by different health care professionals (ie, physiotherapists, general practitioners, and an orthopedic surgeon) partnered with the research consortium. Outreach methods include online and offline leaflets, local newspaper advertisements, engagement with local health care professionals, letters from general practitioners, social media, and promotion via the Dutch Arthritis Society. Individuals who are interested in participating will be able to register via a Castor web-based study portal, phone, or email.

### Intervention

For this study, the e-cOAch version 1 web app was developed in a co-design process with a patient panel and health care professionals to ensure technical functionality, usability, and relevance. The e-cOAch version 1 is a Class IIa medical device under the European Union Medical Device Regulation (2017/745) and is used in this study under the Medical Device Regulation article 82 [20]. The e-cOAch version 1 was primarily designed for data collection, with additional self-care and osteoarthritis knowledge content based on national and international hip and knee osteoarthritis guidelines [5,6,21] (Table 1).

**Table 1.** Summary of the content of the e-cOAch.

Program	Summary content
Start program (duration of 2 weeks)	Eight informational articles cover topics including the nature of osteoarthritis, pain mechanisms, common symptoms, self-management strategies, the importance of exercise and a healthy lifestyle, walking aids, health care professionals, and information about medication use for osteoarthritis. They include text, videos, and assignments. The information is in line with international and national guidelines [5,6,21,22].
Physical activity promotion program (duration of 12 weeks)	The physical activity program builds on previous research done by our team [23-25]. This program aims to increase knowledge and levels of physical activity and improve muscle strength through three components: Weekly education on movement, pain, energy management, and recommended moderate to vigorous physical activity levels A graded activity module with baseline measurement (Fitbit Inspire 3 or self-reported physical activity), goal setting, weekly increases of moderate to vigorous physical activity, and reinforcement Strength exercises provided via instructional videos, performed twice weekly for 20 minutes
Sleep optimization program (duration of 12 weeks)	Aims to improve insomnia severity through three components, in line with insomnia guidelines [26,27]: Weekly education on sleep and sleep hygiene, including recommended behaviors to promote sleep hygiene (eg, relaxation, consistent routines, environment modification, limiting caffeine, and screen time) Behavior change support with goal setting, tailored feedback, and prompts to encourage healthier sleep habits Mindfulness exercises such as progressive muscle relaxation and meditation [28]
Weight management program (duration of 12 weeks)	Supports participants in adopting a healthier diet and achieving a healthier weight if needed: Participants can monitor their weight and receive feedback on their BMI Complete an FFQ^a^ [29] for personalized feedback and select food components to focus on. The FFQ represents the Dutch food–based dietary guidelines [29] Participants receive daily personalized tips and weekly information on healthy eating and weight loss
Advice from a health care professional (duration is one moment of advice)	People with severe complaints (NPRS^b^≥7) for 4 consecutive weeks receive information about relevant professionals. According to Dutch nutritional guidelines [30,31], underweight (BMI<18.5) or extremely overweight people (BMI>30) are advised to visit a dietician. People with high scores of insomnia who have already participated in the sleep program are advised to visit a general practitioner or sleep specialist. Participants with a PAR-Q^c^ score above 0 require medical clearance for the physical activity promotion program and are advised to consult a general practitioner [32].

a FFQ: food frequency questionnaire.

b NPRS: Numeric Pain Rating Score.

c PAR-Q: Physical Activity Readiness Questionnaire.

The e-cOAch offers education on osteoarthritis, self-care advice (ie, physical activity promotion, weight management, and sleep optimization), and guidance on seeking professional care. Content is delivered through text, videos, and interactive assignments (Table 1). Participants who start supervised treatment with a health care professional may continue with the app or pause participation. To minimize dropout and maintain engagement, the study will combine monitoring, timely tailored reminders, and optional personal phone follow-ups. Harms and adverse events will be followed up by phone after they are reported by the participant.

### Outcome Measures and Data Collection

All outcome measures are self-reported via questionnaires in the e-cOAch. To improve inclusivity, all questionnaires are adapted to language level B1. This is in line with the Pharos guideline for understandable questionnaires [33]. Pharos is a Dutch national expertise center on health equity.

The primary outcome is self-reported physical functioning, measured using the Hip Disability and Osteoarthritis Outcome Score (HOOS) [34] and the Knee Injury and Osteoarthritis Outcome Score (KOOS) [35]. Secondary outcomes are pain (Numeric Pain Rating Scale) and participation (Patient-Reported Outcomes Measurement Information System Experience, Short-Form version 8a) [36]. All outcome measures are presented in Table 2. All selected instruments have demonstrated acceptable-to-good reliability and validity. Outcome measures are based on 2 systematic reviews and include known factors that influence physical functioning, weight management, and sleep behavior in osteoarthritis [37,38]. Baseline characteristics (Table 3) will be collected at the start of using the e-cOAch, including digital health literacy. Ethnicity (Asian or non-Asian) is recorded because BMI calculations differ in Asian populations [39].

**Table 2.** Measurement instruments.

Outcomes	Measurement instrument	Measurement properties
Primary outcome		
Physical functioning	The HOOS^a^ [34] or KOOS^b^ [35]	The HOOS includes 40 items, and the KOOS includes 42 items in 5 subdomains of physical functioning. Each item is rated on a 5-point Likert scale. The total scores range from 0 to 100; a higher score represents better physical functioning. Good test-retest reliability, internal consistency, and construct validity in Dutch osteoarthritis populations [34].
Secondary outcomes		
Pain	NRS-11^c^ [40]	The NPRS^d^ ranges from 0 (no pain) to 10 (worst pain imaginable). Moderate-to-good test-retest reliability and acceptable construct validity [40].
Participation	The Patient-Reported Outcomes Measurement Information System Experience, Short-Form version 8a [36]	Consists of 8 items, each rated on a 5-point Likert scale. Higher scores indicate a higher ability to participate in social roles and activities. Excellent internal consistency and good construct validity [36].
Other outcomes		
Stiffness	Subscale of the HOOS [34] or the KOOS [35]	The subscale stiffness consists of 2 items with a 5-point Likert scale. A higher score represents less stiffness. Good test-retest reliability, internal consistency, reliability, and construct validity in Dutch osteoarthritis populations [34,35].
Physical activity (self-reported)	BPAAT^e^ [41]	Two items regarding the frequency and duration of physical activity. The subject can be classified as insufficiently (0‐3 score) or sufficiently active (>3 score). Insufficient refers to not meeting the World Health Organization guideline of 150 minutes of moderate-to-vigorous physical activity per week. Good test-retest reliability and good construct validity [41].
Physical activity (device measured)	The Fitbit Inspire 3 measures physical activity in a random subset (n=200) of the study population	Device-measured active minutes (Active Zone Minutes) will be used to assess if someone is meeting the World Health Organization guideline of 150 minutes of moderate-to-vigorous physical activity per week.
Fatigue	The NRS-11 [40]	One item reports fatigue on a scale from 0 (no fatigue) to 10 (extreme fatigue).
Job satisfaction	The NRS-11 [40]	One item reports job satisfaction on a scale from 0 (not satisfied at all) to 10 (completely satisfied).
Depression and anxiety	HADS^f^ [42]	Fourteen items report anxiety and depression symptoms over the past month on a 4-point Likert scale. A higher score indicates more severe symptoms. Good test-retest reliability, good internal consistency and reliability, and good construct validity [42].
Experienced social support	The NRS-11 [40]	One item reports experienced social support on a scale from 0 (no support) to 10 (extensive support).
Kinesiophobia	BFOM^g^ [43]	Six items that report fear on a 5-point Likert scale from 1 (strongly disagree) to 5 (strongly agree). A higher score indicates greater fear. Good test-retest reliability, good internal consistency and reliability, and acceptable-to-good construct validity [43].
Self-efficacy	A shortened version of the ASES^h^, excluding the function subscale [44].	Eleven items that report confidence in managing arthritis-related symptoms with a 5-point Likert scale. A higher score indicates better self-efficacy. Good test-retest reliability, good internal consistency, and good construct validity [44].
Insomnia severity	The ISI^i^ [45].	Seven items that report insomnia severity on a 5-point Likert scale. A higher score indicates more severe insomnia. Good test-retest reliability, good internal consistency, and good construct validity.
Pain coping	The PCI^j^ [46].	Thirty-three items that report 6 coping styles on a 4-point Likert scale. The highest number indicates the coping style the participant applies most. Good internal consistency and moderate construct validity.
Weight	Will be evaluated with a scale in the participants’ private environment.	—^k^
Physical activity readiness	The PAR-Q^l^ [32].	Seven yes or no questions about health conditions. If any yes answers are given, medical clearance may be needed before starting activity programs. In this study, the question about joint pain is excluded, since all participants will answer yes. Reliability is not applicable, but criterion validity is acceptable [47].
Sleep quality	The PSQI^m^ [48].	Nineteen items that report sleep quality over the past month on a 4-point Likert scale. A higher score indicates poorer sleep quality. Acceptable-to-good test-retest reliability, internal consistency and good criterion validity [48].
Health care utilization	—	One yes or no question that evaluates if participants have received any care in the past month for their osteoarthritis complaints. If they answered yes, participants can choose between a list of health care professionals.
Relevance of advice	—	The relevance of advice in the e-cOAch (one of the programs or consulting a health care professional). One item on a 5-point Likert scale. A higher score indicates more relevant advice.
Timing of advice	—	The relevance of advice in the e-cOAch (one of the programs or consulting a health care professional). One item on a 5-point Likert scale. A higher score indicates better timing of the advice.
Attitude	—	Attitude is reported by 3-6 questions about the target behavior on a 4-point Likert scale. For example, “Do you find it important to [target behavior]?”
Intention	—	Intention is reported by 3 questions about the target behavior on a 4-point Likert scale. For example, “How strong do you plan to [target behavior]?”
Perceived behavioral control	—	Perceived behavioral control is assessed by 4 questions about the target behavior on a 4-point Likert scale. For example, “How much control do you have on [target behavior]?”
Engagement	—	Session start times, session duration, pages visited per session, total number of clicks, reminders that were opened, reminders that led user to start app, interaction with content items, and 5-point Likert scale rating for information in the app.
Digital literacy	—	One question that screens digital literacy on a 5-point Likert scale: “How handy are you with apps?”
Health literacy	—	One question that screens health literacy on a 5-point Likert scale: “How confident are you filling out medical forms by yourself?” Good concurrent criterion validity compared with the reference standard [49].

a HOOS: Hip Disability and Osteoarthritis Outcome Score.

b KOOS: Knee Injury and Osteoarthritis Outcome Score.

c NRS-11: 11-item Numeric Rating Scale.

d NPRS: Numeric Pain Rating Scale.

e BPAAT: Brief Physical Activity Assessment Tool.

f HADS: Hospital Anxiety and Depression Scale.

g BFOM: Brief Fear of Moving.

h ASES: Arthritis Self-Efficacy Scale.

i ISI: Insomnia Severity Index.

j PCI: Pain Coping Inventory.

k Not applicable.

l PAR-Q: Physical Activity Readiness Questionnaire.

m PSQI: Pittsburgh Sleep Quality Index.

**Table 3.** Measurement timepoints.

Week	1	3	5	7	9	11	13	14 until 51^a^	52
Characteristics								^a^	
Date of birth	✓							^a^	
Sex	✓							^a^	
Height	✓							^a^	
Level of education	✓							^a^	
Comorbidity	✓							^a^	✓
Type osteoarthritis	✓							^a^	
Ethnicity (Asian or non-Asian)	✓							^a^	
Use of a walking device	✓							^a^	✓
Use of pain medication	✓							^a^	✓
Device for e-cOAch	✓							^a^	
Duration of osteoarthritis complaints	✓							^a^	
Health literacy	✓							^a^	
Digital literacy	✓							^a^	
Smoking	✓							^a^	
Alcohol use	✓							^a^	
Sleep quality	✓							^a^	
Health care utilization	✓		✓		✓		✓	^a^	✓
Primary outcome									
Physical functioning		✓					✓	^a^	
Secondary outcomes									
Pain	✓	✓	✓	✓	✓	✓	✓	^a^	
Stiffness	✓	✓	✓	✓	✓	✓	✓	^a^	
Physical activity (min)	✓	✓	✓	✓	✓	✓	✓	^a^	
Participation		✓					✓	^a^	
Job satisfaction			✓					^a^	
Depression and anxiety			✓					^a^	
Social support				✓				^a^	
Kinesiophobia				✓				^a^	
Pain coping				✓				^a^	
Physical activity readiness	✓				✓			^a^	
Fatigue					✓			^a^	
Self-efficacy					✓			^a^	
Insomnia severity		✓					✓	^a^	
Weight		✓					✓	^a^	
Self-care program outcomes									
Sleep optimization: insomnia severity^b^									
Weight management: weight^b^									
Physical activity promotion: physical activity^b^									
Attitude, intention, and perceived behavioral control^c^									

a Pattern of the measurements between week 3 and 13 repeats from week 14 until week 51.

b These measurements take place before and after the self-care programs.

c These measurements take place before the self-care programs.

Baseline and follow-up assessments are conducted remotely and distributed over 52 weeks to minimize participant burden. Pain, physical activity, and stiffness are assessed biweekly, while slower-changing outcomes are measured every 10 weeks (Table 3).

### Randomization and Blinding

In total, 18 sequences of the self-care programs are possible due to the restriction that participants cannot end the study with the control period. Participants are assigned to one of the 18 sequences (Table 1, Figure 1). Each sequence consists of three 12-week self-care programs: physical activity promotion, weight management, and sleep optimization, as well as a 12-week control period. Programs start at weeks 3, 15, 27, and 39. Each program is delivered once per participant consecutively, without intermissions.

Randomization is performed at baseline using a computer-generated allocation sequence embedded within the e-cOAch web app, assigning participants with equal probability to one of the 18 intervention sequences. Allocation was computer generated and concealed until disclosure by an independent staff member before baseline assessment.

For safety reasons, extremely underweight participants (BMI<17.5) are not assigned to the weight management program; these participants instead follow a 12-week no-intervention period and are advised to consult a dietitian. Additionally, underweight participants (BMI<18.5), overweight participants (BMI>30), or those requiring medical clearance for physical activity (Physical Activity Readiness Questionnaire >0), are advised to consult a health care professional but remain eligible for all programs.

The study is conducted in an open-label design, as blinding participants and researchers is not feasible given the nature of the self-care programs.

### Sample Size

We aim to recruit 600 participants within 6 months. Data will be split into training and test sets for the development and validation of predictive algorithms. A random subset of the data will be reserved for testing to evaluate model performance on unseen data [50].

Combining multiple predictors and allowing for nonlinear relationships is expected to improve the prediction of pain, physical functioning, and participation [50]. A large sample is therefore required to capture diverse combinations of participant characteristics and support model validation. Following standard practice, 20% to 30% of the data will be used for testing and 70% to 80% for training [50], resulting in an estimated 100 to 150 participants in the test set.

To account for an anticipated 15% dropout [14], we aim to recruit 600 participants, yielding an expected final sample size of approximately 510 participants. Given the high prevalence of osteoarthritis and the broad reach of project partners, this recruitment target is considered feasible.

### Statistical Analysis Plan

#### Overview of Statistical Analysis

The primary objective of this study is to develop and evaluate data-driven models that support personalized recommendations on the optimal timing and optimal advice (physical activity promotion, sleep optimization, and weight management) for individuals with hip or knee osteoarthritis. In other words, the study aims to identify a dynamic treatment regime. To achieve this objective, the following steps will be taken to develop and evaluate 2 data-driven models.

#### Step A: Data Preparation and Missing Data

Before model development, data completeness and patterns of missingness will be examined. Missing data are expected due to the longitudinal design and repeated measurements. All analyses will include all randomized participants and will follow the intention-to-treat principle. When appropriate, missing values will be handled using multiple imputation by chained equations under a missing-at-random assumption. Sensitivity analyses will be conducted to assess the robustness of results to alternative missing-data assumptions. Information on reasons for dropout will be used to support assumptions regarding missing data mechanisms. Time to dropout will additionally be considered a process in longitudinal analyses where relevant. To obtain a reliable estimate of model performance and uncertainty, we will use cross-validation and bootstrapping as recommended in the TRIPOD + AI statement [51].

#### Step B: Prediction of Deterioration Over Time (Optimal Timing)

Prediction models will be developed to identify individuals at risk of deterioration in physical functioning (primary outcome) and pain and participation (secondary outcomes), over 12 weeks.

Deterioration is operationalized as a decrease in the HOOS subscale activities of daily living (ADL) of 6.7 [52] or the KOOS subscale ADL of 8.2 [53] (in line with the minimal important change). The HOOS and KOOS ADL subscales were selected as the primary outcome because they are disease-specific, sensitive to meaningful change, and directly reflect physical functioning relevant to daily life in people with hip or knee osteoarthritis. The appropriateness of these thresholds will be evaluated as part of a sensitivity analysis by testing alternative cut-off values and assessing their impact on predictive performance.

Supervised machine learning models will be trained using longitudinal data and relevant characteristics, including demographics and physical functioning at baseline. Internal validation will be performed using resampling techniques, including cross-validation and bootstrapping, to assess model performance in terms of calibration and discrimination. Model development and reporting will follow the TRIPOD + AI guidelines for prediction modeling studies [51].

#### Step C: Estimating the Effectiveness of Self-Care Programs (Optimal Advice)

To determine which self-care program is most beneficial for individual patients, causal machine learning methods will be applied, leveraging the randomized cross-over design and the longitudinal data structure.

To support personalized self-care, the causal effects of each self-care program will be estimated using program-specific outcomes that directly reflect the intended targets of the interventions. Specifically, the causal effect of the physical activity promotion program on meeting the physical activity guideline (≥150 minutes of moderate to vigorous intensity physical activity per week; yes/no), the weight management program on body weight (kg), and the sleep optimization program on improvement in insomnia severity will be evaluated. Together, the estimated effects of each program will allow us to determine the optimal dynamic treatment regime.

Causal effects will be estimated using doubly robust methods, including longitudinal targeted maximum likelihood estimation [54,55] and classification-based approaches such as support vector machines and regression trees. These methods can account for time-varying treatments and treatment-confounder feedback inherent to the longitudinal and cross-over design [55]. An ongoing literature review informs the final selection of methods for estimating causal effects. To address potential period and carryover effects inherent to the cross-over design, models will include time and period indicators, and sensitivity analyses will be conducted to assess the robustness of estimated causal effects.

Heterogeneous treatment effects will be explored by evaluating effect modification across predefined subgroups based on psychosocial and behavioral predictors identified in prior literature [38,56].

By focusing on program-specific outcomes, these analyses provide actionable information on which self-care program is most likely to be effective for an individual at a given time point. Downstream effects on physical functioning, pain, and participation will be explored in subsequent analyses. Detailed reporting on model building steps, parameter tuning, validation, and the comparison of multiple models will be provided.

#### Step D: Use of Wearable Data

In a subset of participants, wearable-derived physical activity data will be collected and used as time-varying covariates in the causal analyses. Moderate-to-vigorous physical activity will be approximated using the Fitbit-derived metric “Active Zone Minutes,” which reflects time spent in activity above personalized heart-rate thresholds.

#### Step E: Model Validation and Robustness

Internal validation will be performed using resampling techniques to estimate model performance and uncertainty. Sensitivity analyses will be conducted to assess the impact of missing data and modeling assumptions on both predictive and causal estimates. Model code, analysis scripts, and model versions will be managed using a Git-based workflow to ensure transparency and reproducibility.

### Ethical Considerations

This study has been reviewed and approved by the NedMec Medical Ethical Committee under the following reference: NL87119.041.24. The study will be conducted according to the principles of the Declaration of Helsinki (version 13) and following the Medical Research Involving Human Subjects Act. Furthermore, the study will adhere to the General Data Protection Regulation to ensure the protection of personal data and the Medical Device Regulation where applicable. The University Medical Center Utrecht is the sponsor of this study and can be contacted through the corresponding author. Any protocol deviations will be communicated to relevant parties. Informed consent will be obtained digitally via Castor eConsent. After registration, individuals will receive detailed study information. After at least 1 week, they will be contacted by telephone to confirm eligibility and understanding before providing consent. Eligible participants will be asked to sign the consent form digitally. Paper consent will be available upon request. Participants can opt out of the study at any moment by contacting the research staff through phone or email.

### Reflexivity and Positionality

Reflexivity enhances transparency and rigor in research by prompting researchers to acknowledge how their assumptions and positionalities influence design, analysis, and interpretation [57]. This study is conducted within a multidisciplinary consortium including specialists in behavior change, psychology, vulnerable groups, computer science, and physiotherapy, whose diverse professional lenses shape our design choices and interpretations. Guided by a pragmatic epistemological stance, we view knowledge as generated through the combined insights of researchers, participants, and their real-world context [58]. To counterbalance disciplinary assumptions, individuals with lived experience of osteoarthritis are acting as consultative contributors: they reviewed the protocol during its development and may collaborate with the research team during the interpretation of the findings. Their involvement and ongoing reflexive team discussions can improve the research quality by challenging researcher assumptions and strengthening interpretation [59].

## Results

The funding for the study was granted in 2023. At the time of manuscript submission, 520 participants had been recruited. Recruitment is expected to be completed in March 2026, with data collection projected to conclude in April 2027. The publication of the results is anticipated in spring 2028.

## Discussion

This study aims to develop data-driven models to support personalized recommendations regarding the optimal timing of self-care programs for individuals with hip or knee osteoarthritis.

Existing digital osteoarthritis self-management tools are typically limited in duration and focus primarily on education and short-term symptom management [14,60,61]. In contrast, this study develops data-driven models to forecast symptom changes and deliver personalized, just-in-time self-management advice, which may improve outcomes by providing support when it is most useful [62].

A key strength of this study is its inclusive design, which facilitates accessibility for individuals with varying levels of digital health literacy. These groups are often underrepresented in digital health research and require additional support to participate [63-65]. We will therefore adjust our recruitment to target these groups by personal contact through health care professionals. Also, all content and questionnaires were adapted to the language level B1. This is in line with the Pharos guideline for understandable questionnaires [33]. We test whether the translated questionnaires retain the validity of the original questionnaires in a separate ongoing study.

Although this study explicitly aims to include individuals with different levels of digital health literacy, it was screened using a brief 2-item screener with a predefined cutoff to limit participant burden in this large, longitudinal study. This pragmatic choice was made in the context of considerable heterogeneity in existing digital and eHealth literacy instruments, for which no universally accepted gold standard currently exists [66,67]. Rather than conducting a comprehensive multidimensional assessment, this approach was intended to provide a pragmatic indication of participants’ affinity with smartphone apps and their fit with the e-cOAch app. As digital health literacy is a multidimensional construct, this approach may not fully capture all relevant skills and could result in some misclassification. This limitation should be considered when interpreting subgroup analyses, and future studies may benefit from using validated digital health literacy measures where feasible.

Maintaining participant engagement is a well-known challenge in digital health studies, with pooled dropout rates of 43% in digital health interventions for chronic diseases [68]. High dropout rates may contribute to data absenteeism, bias AI models, and reduce generalizability [65,69]. To minimize dropout and maintain engagement, the study combines monitoring, timely tailored reminders, periodic study newsletters providing updates, optional personal phone follow-ups, incentives for achieving certain completion rates, and program personalization [14]. The patient panel tested e-cOAch version 1, and their feedback was used to refine the app’s usability, readability, and navigability [70]. This approach supports the development of AI models for e-cOAch and other digital self-management tools that are representative of a broad population.

A limitation is that while we aim to identify predictors of changes in physical functioning, these outcomes will also be influenced by the self-care programs participants receive. Therefore, associations between measurements may reflect both underlying prognostic factors and the effects of self-care programs. In addition, physical functioning in osteoarthritis is influenced by many factors [37,38], making it difficult to capture in a dataset. The results should be interpreted with caution, as predictive associations may reflect intervention effects, and validation in populations not receiving self-care advice may be needed.

This study will deliver data-driven models to support personalized recommendations on the optimal timing of self-care programs (physical activity, sleep, and weight management) for individuals with hip or knee osteoarthritis. These models will be integrated into a new iteration of e-cOAch (version 2) and evaluated in a future effectiveness study. In addition, the data from this cross-over study will address several further research questions. First, can distinct subgroups of individuals with hip or knee osteoarthritis be identified based on determinants of self-management behavior? Second, what is the course of symptom flare-ups in people with hip and knee osteoarthritis, and which factors predict their occurrence over time? Third, how do individuals with hip or knee osteoarthritis score on participation, and which personal or contextual factors are associated with participation outcomes? Finally, how do people with hip and/or knee osteoarthritis sleep and what is the association between weight, physical activity, pain, and quality of life?

## Supplementary material

10.2196/94709 Peer Review Report 1 Peer review report from the Dutch Research Council (NWO).

## Abbreviations

ADL: activities of daily living
AI: artificial intelligence
HOOS: Hip Disability and Osteoarthritis Outcome Score
KOOS: Knee Injury and Osteoarthritis Outcome Score
SPIRIT: Standard Protocol Items: Recommendations for Interventional Trials
TRIPOD: Transparent Reporting of a Multivariable Prediction Model for Individual Prognosis or Diagnosis

## References

[1] Steinmetz JD, Culbreth GT, Haile LM, GBD 2021 Osteoarthritis Collaborators (2023). Global, regional, and national burden of osteoarthritis, 1990–2020 and projections to 2050: a systematic analysis for the Global Burden of Disease Study 2021. Lancet Rheumatol.

[2] Hunter DJ, Bierma-Zeinstra S (2019). Osteoarthritis. Lancet.

[3] Wallis JA, Taylor NF, Bunzli S, Shields N (2019). Experience of living with knee osteoarthritis: a systematic review of qualitative studies. BMJ Open.

[4] Bieleman HJ, Oosterveld FGJ, Oostveen JCM, Reneman MF, Groothoff JW (2010). Work participation and health status in early osteoarthritis of the hip and/or knee: a comparison between the Cohort Hip and Cohort Knee and the Osteoarthritis Initiative. Arthritis Care Res.

[5] (2018). KNGF-richtlijn artrose heup-knie: conservatieve, pre- en postoperatieve behandeling [Report in Dutch]. https://reumanetnl.nl/wp-content/uploads/2019/06/KNGF-praktijkrichtlijn-Artrose-heup-knie-2018.pdf.

[6] Belo JN, Bierma-Zeinstra SMA, Kuijpers T (2016). NHG-standaard niet-traumatische knieklachten [Article in Dutch]. Huisarts Wet.

[7] Wu Z, Zhou R, Zhu Y (2022). Self‐management for knee osteoarthritis: a systematic review and meta‐analysis of randomized controlled trials. Pain Res Manag.

[8] Smink AJ, van den Ende CHM, Vliet Vlieland TPM (2011). “Beating osteoARThritis”: development of a stepped care strategy to optimize utilization and timing of non-surgical treatment modalities for patients with hip or knee osteoarthritis. Clin Rheumatol.

[9] (2025). Stepped care en regionale verschillen in artrosezorg: een analyse van de zorg voor knie- en heupartrose in de huisartsenzorg, fysiotherapie en orthopedie [Report in Dutch]. https://fysionieuws.nl/wp-content/uploads/2025/08/Steppedcare.pdf.

[10] (2022). Osteoarthritis in over 16s: diagnosis and management. National Institute for Health and Care Excellence.

[11] Moseng T, Vliet Vlieland TPM, Battista S (2024). EULAR recommendations for the non-pharmacological core management of hip and knee osteoarthritis: 2023 update. Ann Rheum Dis.

[12] Shah N, Costello K, Mehta A, Kumar D (2022). Applications of digital health technologies in knee osteoarthritis: narrative review. JMIR Rehabil Assist Technol.

[13] Safari R, Jackson J, Sheffield D (2020). Digital self-management interventions for people with osteoarthritis: systematic review with meta-analysis. J Med Internet Res.

[14] Patten RK, Tacey A, Pile R (2022). Digital self-management interventions for osteoarthritis: a systematic scoping review of intervention characteristics, adherence and attrition. Arch Public Health.

[15] Alkhuzaimi F, Rainey D, Brown Wilson C, Bloomfield J (2025). The impact of mobile health interventions on service users’ health outcomes and the role of health professions: a systematic review of systematic reviews. BMC Digit Health.

[16] Matheny M, Israni ST, Whicher D, Ahmed M (2022). Artificial Intelligence in Health Care: The Hope, the Hype, the Promise, the Peril.

[17] Kulnik ST, Carrozzo AE, Crutzen R (2025). Persistent carry-over in a two-period randomised crossover design for behavioural interventions without the expectation of return to baseline after intervention cessation. Trials.

[18] Shi D, Ye T (2024). Behavioral carry-over effect and power consideration in crossover trials. Biometrics.

[19] Chan AW, Boutron I, Hopewell S (2025). SPIRIT 2025 statement: updated guideline for protocols of randomised trials. BMJ.

[20] (2017). Regulation (EU) 2017/745 of the European Parliament and of the Council of 5 April 2017 on medical devices, amending Directive 2001/83/EC, Regulation (EC) no 178/2002 and Regulation (EC) no 1223/2009 and repealing Council Directives 90/385/EEC and 93/42/EEC (Text with EEA relevance). http://data.europa.eu/eli/reg/2017/745/oj.

[21] Bannuru RR, Osani MC, Vaysbrot EE (2019). OARSI guidelines for the non-surgical management of knee, hip, and polyarticular osteoarthritis. Osteoarthr Cartil.

[22] French SD, Bennell KL, Nicolson PJA, Hodges PW, Dobson FL, Hinman RS (2015). What do people with knee or hip osteoarthritis need to know? An international consensus list of essential statements for osteoarthritis. Arthritis Care Res (Hoboken).

[23] Kloek CJJ, Bossen D, Spreeuwenberg PM, Dekker J, de Bakker DH, Veenhof C (2018). Effectiveness of a blended physical therapist intervention in people with hip osteoarthritis, knee osteoarthritis, or both: a cluster-randomized controlled trial. Phys Ther.

[24] Bossen D, Veenhof C, Van Beek KE, Spreeuwenberg PM, Dekker J, De Bakker DH (2013). Effectiveness of a web-based physical activity intervention in patients with knee and/or hip osteoarthritis: randomized controlled trial. J Med Internet Res.

[25] Veenhof C, Köke AJA, Dekker J (2006). Effectiveness of behavioral graded activity in patients with osteoarthritis of the hip and/or knee: a randomized clinical trial. Arthritis Rheum.

[26] Hwang JW, Lee GE, Woo JH, Kim SM, Kwon JY (2025). Systematic review and meta-analysis on fully automated digital cognitive behavioral therapy for insomnia. NPJ Digit Med.

[27] Riemann D, Espie CA, Altena E (2023). The European Insomnia Guideline: an update on the diagnosis and treatment of insomnia 2023. J Sleep Res.

[28] Rash JA, Kavanagh VAJ, Garland SN (2019). A meta-analysis of mindfulness-based therapies for insomnia and sleep disturbance: moving towards processes of change. Sleep Med Clin.

[29] de Rijk MG, Slotegraaf AI, Brouwer-Brolsma EM, Perenboom CWM, Feskens EJM, de Vries JHM (2021). Development and evaluation of a diet quality screener to assess adherence to the Dutch food-based dietary guidelines. Br J Nutr.

[30] (2019). Richtlijn ondervoeding: herkenning, diagnosestelling en behandeling van ondervoeding bij volwassenen [Report in Dutch]. https://www.kenniscentrumondervoeding.nl/wp-content/uploads/2022/04/SoV01-Richtlijn-Ondervoeding-februari-2019-met-addendum-september-2021.pdf.

[31] van Binsbergen JJ, Langens FNM, Dapper ALM (2010). NHG-Standaard M95. NHG-Standaard Obesitas. Huisarts Wet.

[32] Warburton DER, Gledhill N, Jamnik VK (2011). Evidence-based risk assessment and recommendations for physical activity clearance: Consensus Document 2011. Appl Physiol Nutr Metab.

[33] (2022). Begrijpelijke vragenlijsten - de basis voor goede zorg [Report in Dutch]. https://www.pharos.nl/infosheets/begrijpelijke-vragenlijsten-de-basis-voor-goede-zorg/.

[34] de Groot IB, Reijman M, Terwee CB (2007). Validation of the Dutch version of the Hip disability and Osteoarthritis Outcome Score. Osteoarthr Cartil.

[35] de Groot IB, Favejee MM, Reijman M, Verhaar JAN, Terwee CB (2008). The Dutch version of the knee injury and osteoarthritis outcome score: a validation study. Health Qual Life Outcomes.

[36] Bode RK, Hahn EA, DeVellis R, Cella D, Patient-Reported Outcomes Measurement Information System Social Domain Working Group (2010). Measuring participation: the patient-reported outcomes measurement information system experience. Arch Phys Med Rehabil.

[37] Paans‐Cijs B, Stekelenburg R, Veenhof C (2025). Prognostic factors and changes in pain, physical functioning, and participation in patients with hip and/or knee osteoarthritis: a systematic review. Arthritis Care Res (Hoboken).

[38] van Dongen B, Ronteltap A, Cijs B, Kloek C, Bolman C, Crutzen R (2025). Psychosocial factors associated with physical activity, weight management, and sleep in adults with hip and knee osteoarthritis: a systematic review. BMC Rheumatol.

[39] Caleyachetty R, Barber TM, Mohammed NI (2021). Ethnicity-specific BMI cutoffs for obesity based on type 2 diabetes risk in England: a population-based cohort study. Lancet Diabetes Endocrinol.

[40] Goldsmith ES, Taylor BC, Greer N (2018). Focused evidence review: psychometric properties of patient-reported outcome measures for chronic musculoskeletal pain. J Gen Intern Med.

[41] Vilarinho R, Amorim L, Gomes D (2025). Validation of the brief physical activity assessment tool: comparison of telephone and in-person administration. PLoS One.

[42] Spinhoven P, Ormel J, Sloekers PP, Kempen GI, Speckens AE, Van Hemert AM (1997). A validation study of the Hospital Anxiety and Depression Scale (HADS) in different groups of Dutch subjects. Psychol Med.

[43] Shelby RA, Somers TJ, Keefe FJ (2012). Brief fear of movement scale for osteoarthritis. Arthritis Care Res.

[44] Lorig K, Chastain RL, Ung E, Shoor S, Holman HR (1989). Development and evaluation of a scale to measure perceived self-efficacy in people with arthritis. Arthritis Rheum.

[45] Morin CM, Belleville G, Bélanger L, Ivers H (2011). The insomnia severity index: psychometric indicators to detect insomnia cases and evaluate treatment response. Sleep.

[46] Kraaimaat FW, Evers AWM (2003). Pain-coping strategies in chronic pain patients: psychometric characteristics of the pain-coping inventory (PCI). Int J Behav Med.

[47] Maranhao Neto GA, Luz LGO, Farinatti PTV (2013). Diagnostic accuracy of pre-exercise screening questionnaire: emphasis on educational level and cognitive status. Arch Gerontol Geriatr.

[48] Buysse DJ, Reynolds CF, Monk TH, Berman SR, Kupfer DJ (1989). The Pittsburgh Sleep Quality Index: a new instrument for psychiatric practice and research. Psychiatry Res.

[49] Chew LD, Griffin JM, Partin MR (2008). Validation of screening questions for limited health literacy in a large VA outpatient population. J Gen Intern Med.

[50] James G, Witten D, Hastie T, Tibshirani R (2021). An Introduction to Statistical Learning.

[51] Collins GS, Moons KGM, Dhiman P (2024). TRIPOD+AI statement: updated guidance for reporting clinical prediction models that use regression or machine learning methods. BMJ.

[52] Chéret EPBM, Mechlenburg I, Skou ST, Dalgas U, Stisen MG, Kjeldsen T (2025). Minimal important change in the hip disability and osteoarthritis outcome score and the European quality of life 5 dimensions in adults with hip osteoarthritis after 12 weeks of exercise. Musculoskelet Sci Pract.

[53] Silva MDC, Perriman DM, Fearon AM, Couldrick JM, Scarvell JM (2023). Minimal important change and difference for knee osteoarthritis outcome measurement tools after non-surgical interventions: a systematic review. BMJ Open.

[54] Schomaker M, Luque-Fernandez MA, Leroy V, Davies MA (2019). Using longitudinal targeted maximum likelihood estimation in complex settings with dynamic interventions. Stat Med.

[55] Hernán MA, Robins JM (2020). Causal Inference: What If.

[56] Zhu J, Gallego B (2020). Targeted estimation of heterogeneous treatment effect in observational survival analysis. J Biomed Inform.

[57] Jamieson MK, Govaart GH, Pownall M (2023). Reflexivity in quantitative research: a rationale and beginner’s guide. Social Personality Psych.

[58] Creswell JW, Poth C (2017). Qualitative Inquiry & Research Design: Choosing among Five Approaches.

[59] Vinnicombe S, Bianchim MS, Noyes J (2023). A review of reviews exploring patient and public involvement in population health research and development of tools containing best practice guidance. BMC Public Health.

[60] My Joint Pain.

[61] OA Coach Study. Osteoarthritis Clinical Research Group.

[62] Du Y, Yang P, Liu Y, Deng C, Li X (2025). Artificial intelligence in chronic disease self-management: current applications and future directions. Front Public Health.

[63] Smith SG, O’Conor R, Aitken W, Curtis LM, Wolf MS, Goel MS (2015). Disparities in registration and use of an online patient portal among older adults: findings from the LitCog cohort. J Am Med Inform Assoc.

[64] Hernandez-Ramos R, Aguilera A, Garcia F (2021). Conducting internet-based visits for onboarding populations with limited digital literacy to an mHealth intervention: development of a patient-centered approach. JMIR Form Res.

[65] Bao H, Wong YJ, Singh NB (2025). Data absenteeism in digital health technology research for older adults: a systematic review. BMC Digit Health.

[66] Lee J, Lee EH, Chae D (2021). eHealth literacy instruments: systematic review of measurement properties. J Med Internet Res.

[67] Huang YQ, Liu L, Goodarzi Z, Watt JA (2023). Diagnostic accuracy of eHealth literacy measurement tools in older adults: a systematic review. BMC Geriatr.

[68] Meyerowitz-Katz G, Ravi S, Arnolda L, Feng X, Maberly G, Astell-Burt T (2020). Rates of attrition and dropout in app-based interventions for chronic disease: systematic review and meta-analysis. J Med Internet Res.

[69] Lee EWJ, Viswanath K (2020). Big data in context: addressing the twin perils of data absenteeism and chauvinism in the context of health disparities research. J Med Internet Res.

[70] Lee EW, McCloud RF, Viswanath K (2022). Designing effective eHealth interventions for underserved groups: five lessons from a decade of eHealth intervention design and deployment. J Med Internet Res.

[71] Public DMPs. DMPonline.

