# Supplementary material for: Development of Data-Driven Models for Just-in-Time Digital Self-Management Advice to Improve Physical Functioning in Hip and Knee Osteoarthritis: Protocol for the e-cOAch Cross-Over Study
Source: JMIR Res Protoc. 2026 Jun 22;15:e94709. doi: 10.2196/94709 (PMC13286329; doi:10.2196/94709)
Supplement: Peer Review Report 1 [file resprot-v15-e94709-s001.pdf]

APPENDIX: ASSESSMENT REPORT

Financing: KIC Missie call Zorg in eigen
leefomgeving Number of project: KICH1.GZ03.2 1.005
Applier: Prof. dr. C. Veenhof
Title project: SMART: development and evaluation of an AI-supported just-in-time adaptive lifestyle app to improve self-management in people with hip and knee osteoarthritis

How was your application assessed?
The assessment committee assessed your application, as it did all other applications, in accordance with the procedure and criteria set out in the Call for Proposals. Your application was given a numerical score for each criterion. The assessment committee then drew up a recommendation. Based on this recommendation, the board decided whether to approve or reject applications.

In this round, NWO used the scoring scale below and the corresponding translation into qualifications. An application must have at least the qualification 'very good' and a score of at least 4.0 for each criterion in order to be eligible for approval.

Score	Kwalification
1,0 t/m 1,4	Excellent
1,5 t/m 3,4	Very Good
3,5 t/m 5,4	Good
5,5 t/m 9,0	Insufficient

Your application has received the weighted final score and qualification shown in the table below.

The scores given have led to a qualitative prioritization of the applications. In total, NWO has selected the six highest-priority applications from the 13 detailed applications submitted.

The substantive assessment of your application
Below you will find an explanation of the assessment committee's opinion for each assessment criterion.

Assessment criteria	
1. Problem definition and analysis, contribution to solution (weighting 25%)	1,70
2. Problem definition and analysis, contribution to solution (weighting 25%)	2,13
3. Quality consortium (weighting 30%)	1,84
4. Quality onderzoek (weighting 20%)	1,84
Endscore	1,80
Qualification	Very good
Place in priority	3

Motivation from the assessment committee:

Problem definition and analysis, contribution to solution (weighting 25%)
The committee believes that both the social and scientific relevance are convincingly and well described. Osteoarthritis of the hip and knees is a relevant social problem that will only increase as the population ages. The problem statement is well defined and the response provides a well-argued case for the involvement of high-performance computing.

The Dutch feedback has been translated into English using DeepL. The first two pages present the English version of the report, followed by the original Dutch version on the subsequent two pages.
For questions or clarification, please contact the corresponding author through f.groen-4@umcutrecht.nl.

Expected impact and route to impact

The committee considers the potential impact to be significant. According to the committee, relevant stakeholders are well involved in the proposal. The social impact is reflected both in the empowerment of people with osteoarthritis and in the reduction of healthcare costs associated with this condition. End users are involved in the development of the technology through co-creation, which increases the potential impact of the project for the target group. In addition, specific attention will be paid to people with low digital and health skills, which will maximize the impact on different socioeconomic groups.

Quality consortium

The interdisciplinary consortium represents expertise from a wide range of relevant disciplines, and the committee believes that the consortium partners complement each other well in this regard. In addition, the project involves several co-financiers and collaboration partners, each of which has a clear role in the project. The field labs provide the link to existing learning communities, which contributes to the promotion of human capital. The committee notes that the elaboration of this is limited.

Quality of research

The committee believes that the research questions are clearly formulated for each phase and each work package. Research activities and methods are described in detail and are appropriate to the research problem. The links with existing field labs and building on existing technology contribute to the feasibility of the proposal. The committee noted with satisfaction the mitigation strategies identified for various important risks that may be encountered within the project. One point of attention raised by the committee concerns data governance, which currently lies with the patient. Instruction and awareness of data governance among patients are therefore important.

The Dutch feedback has been translated into English using DeepL. The first two pages of this document present the English version of the report, followed by the original Dutch version on the subsequent two pages.
For questions or clarification, please contact the corresponding author through f.groen-4@umcutrecht.nl.

BIJLAGE: BEOORDELINGSRAPPORT

Financiering: KIC Missie call Zorg in eigen leefomgeving
Dossiernummer: KICH1.GZ03.2 1.005
Aanvrager: Prof. dr. C. Veenhof
Titel project: SMART: development and evaluation of an AI-supported just-in-time adaptive lifestyle app to improve self-management in people with hip and knee osteoarthritis

Hoe is de beoordeling van uw aanvraag tot stand gekomen?
De beoordelingscommissie heeft uw bovenvermelde aanvraag, gelijk aan alle andere aanvragen, beoordeeld volgens de procedure en de criteria uit de Call for proposals. Per criterium is uw aanvraag voorzien van een cijfermatige score. Vervolgens heeft de beoordelingscommissie een advies opgesteld. Op basis van het advies heeft het bestuur tot toewijzing of afwijzing van aanvragen besloten.

NWO hanteert in deze ronde de onderstaande scoreschaal en de bijbehorende vertaalslag naar kwalificaties. Een aanvraag moet ten minste de kwalificatie 'zeer goed' hebben, alsmede per criterium ten minste de score 4,0 om in aanmerking te kunnen komen voor toewijzing.

Score	Kwalificatie
1,0 t/m 1,4	Excellent
1,5 t/m 3,4	Zeer goed
3,5 t/m 5,4	Goed
5,5 t/m 9,0	Ontoereikend

Uw aanvraag heeft de in onderstaande tabel genormaliseerde gewogen eindscore en kwalificatie ontvangen.

De gegeven scores hebben geleid tot een kwalitatieve prioritering van de aanvragen. In totaal heeft NWO de 6 hoogst geprioriteerde aanvragen van de in totaal 13 ingediende uitgewerkte aanvragen.

De inhoudelijke beoordeling van uw aanvraag
Hieronder vindt u per beoordelingscriterium een uiteenzetting van het oordeel van de beoordelingscommissie.

Beoordelingscriteria	
1. Probleemstelling en —analyse, bijdrage aan oplossing (weging 25%)	1,70
2. Probleemstelling en —analyse, bijdrage aan oplossing (weging 25%)	2,13
3. Kwaliteit consortium (weging 30%)	1,84
4. Kwaliteit onderzoek (weging 20%)	1,84
Eindscore	1,80
Kwalificatie	Zeer goed
Plaats in de prioritering	3

Motivering van de beoordelingscommissie:

Probleemstelling en analyse bijdrage aan oplossing
De commissie is van mening dat zowel de maatschappelijke als de wetenschappelijke relevantie overtuigend en goed beschreven zijn. Artrose aan de heup en knieën is een relevant maatschappelijk probleem wat door de vergrijzing alleen maar zal toenemen. De probleemstelling is goed gedefinieerd en in het weerwoord wordt de betrokkenheid van *high performance computing* goed beargumenteerd.

Verwachte impact en route naar impact
De commissie acht de potentiële impact groot. Relevante stakeholders zijn volgens de commissie goed betrokken in het voorstel. De maatschappelijke impact uit zich zowel op het vlak van *empowerment* van mensen met artrose alsook op het verminderen van de zorgkosten in verband met deze aandoening. Eindgebruikers worden betrokken bij de ontwikkeling van de technologie door middel van co-creatie, wat de potentiële impact van het project voor de doelgroep vergroot. Bovendien zal er specifiek aandacht worden besteed aan mensen met lage digitale vaardigheden en gezondheidsvaardigheden, wat de impact op verschillende sociaaleconomische groepen zal maximaliseren.

Kwaliteit consortium
Het interdisciplinaire consortium vertegenwoordigt expertise uit een breed scala aan relevante disciplines en de commissie is van mening dat de consortiumpartners hierin goed complementair zijn. Daarnaast zijn er bij het project meerdere CO-financiers en samenwerkingspartners betrokken die elk een duidelijke rol hebben in het project. De *fieldlabs* zorgen voor de koppeling met bestaande leergemeenschappen, wat bijdraagt aan het bevorderen van *human capital.* De commissie merkt op dat de uitwerking hiervan beperkt is.

Kwaliteit onderzoek
De commissie is van mening dat de onderzoeksvragen helder zijn verwoord per fase en per werkpakket. Onderzoeksactiviteiten en methoden worden gedetailleerd beschreven en passen bij het onderzoeksprobleem. De koppelingen met bestaande *fieldlabs* en het voortbouwen op bestaande technologie draagt bij aan de haalbaarheid van het voorstel. De commissie nam met tevredenheid kennis van de mitigatiestrategieën zoals geïdentificeerd voor verschillende belangrijke risico's die men binnen het project kan tegenkomen. Een aandachtspunt dat de commissie meegeeft betreft de dataregie, die nu namelijk bij de patiënt ligt. Instructie en bewustwording van dataregie bij de patiënt zijn dus belangrijk.
